# Review of Biomechanical Studies and Finite Element Modeling of Sternal Closure Using Bio-Active Adhesives

**DOI:** 10.3390/bioengineering9050198

**Published:** 2022-05-03

**Authors:** Amatulraheem Al-Abassi, Marcello Papini, Mark Towler

**Affiliations:** 1 Department of Biomedical Engineering, Ryerson University, Toronto, ON M5B 2K3, Canada; mpapini@ryerson.ca (M.P.); mtowler@ryerson.ca (M.T.); 2 Li Ka Shing Knowledge Institute, St. Michael’s Hospital, Toronto, ON M5B 1W8, Canada; 3 Department of Mechanical Engineering, Ryerson University, Toronto, ON M5B 2K3, Canada

**Keywords:** sternum, median sternotomy, sternal fixation, finite element analysis, biomechanical modeling

## Abstract

The most common complication of median sternotomy surgery is sternum re-separation after sternal fixation, which leads to high rates of morbidity and mortality. The adhered sternal fixation technique comprises the wiring fixation technique and the use of bio-adhesives. Adhered sternal fixation techniques have not been extensively studied using finite element analysis, so mechanical testing studies and finite element analysis of sternal fixation will be presented in this review to find the optimum techniques for simulating sternal fixation with adhesives. The optimal wiring technique should enhance bone stability and limit sternal displacement. Bio-adhesives have been proposed to support sternal fixation, as wiring is prone to failure in cases of post-operative problems. The aim of this paper is to review and present the existing numerical and biomechanical sternal fixation studies by reviewing common sternal closure techniques, adhesives for sternal closure, biomechanical modeling of sternal fixation, and finite element modeling of sternal fixation systems. Investigating the physical behavior of 3D sternal fixation models by finite element analysis (FEA) will lower the expense of conducting clinical trials. This indicates that FEA studies of sternal fixation with adhesives are needed to analyze the efficiency of this sternal closure technique virtually.

## 1. Introduction

Median sternotomies are the most frequently performed open cardiac surgeries [1], with over 1 million cardiac operations and more than 1.5 million sternotomy operations performed worldwide [2,3]. In order to access the heart and surrounding organs, the sternum is opened through a vertical inline incision [4,5,6]. 

Post sternotomy complications can include sternal infections, which increase treatment costs and cause longer hospital stays and increased morbidity and mortality [7]. In 2014, cardiovascular disease cost an estimated USD 200 billion [8], and more than USD 3 billion of this was spent directly on treating sternotomy medical-related conditions [9]. Moreover, Awad et al. [2] reported that mortality and morbidity complications increased in adult patients who had a second sternotomy for heart transplantation compared to patients who had one prior sternotomy. Postoperatively, patients who underwent prior sternotomy had longer ICU stays and a higher chance of operational bleeding than patients who had not undergone prior sternotomy [2]. In addition, Vestergaard et al. [3] reported that other complications might arise after a median sternotomy; these include sternal separation, chronic inflammation in the thorax system, bleeding out of the sternotomy, and subcutaneous infection. 

Because sternal re-separation is one of the main surgical complications [9], surgeons have explored new techniques to precisely close the two halves of the sternum. Thus, there is a need for a safer fixation technique that can overcome post-operational complications and facilitate the healing process. 

There are several surgical techniques used to close a median sternotomy. Plass et al. [10] stated that Milton was the first surgeon who performed a median sternotomy using wires for sternum fixation in 1897 [10]. Currently, stainless steel wires or clips are commonly used for sternal closures [11]. These techniques are effective in healthy patients; however, patients who are weak, elderly, or have osteoporosis are more prone to complications when wiring is used [12]. Many wiring techniques have been used as sternal fixation treatments. Modified longitudinal parasternal wiring is a closure technique that has been proven to be safer, more effective, and more economical for patients with a high risk of sternal dehiscence [11]. In addition, adhesives have been used in conjunction with wiring techniques for sternal fixation [13].

Regular wiring treatment methods are associated with instability, which leads to post-operative complications [14]. As a result of wiring instability, median sternotomy complications may cause pain, difficulty breathing, and mediastinitis [15]. Thus, relying solely on wiring may not be the best treatment for sternal fixation [15]. According to Clarkin and Towler, adhesives exhibit potential mechanical strength and handling properties in luting applications [16]. Hence, adhesives have been used to support wiring in clinical trials. However, Kobayashi et al. [17] found that some formations of calcium sodium phosphosilicate putty (NovaBone, LLC) caused an inflammatory reaction throughout the bone formation process and also showed biocompatible resorption and evidence of osteoconduction [17]. Therefore, there is an essential need to study the impact of different bioactive materials and their mechanical properties on various sternotomy closure techniques. 

**Figure 1:**
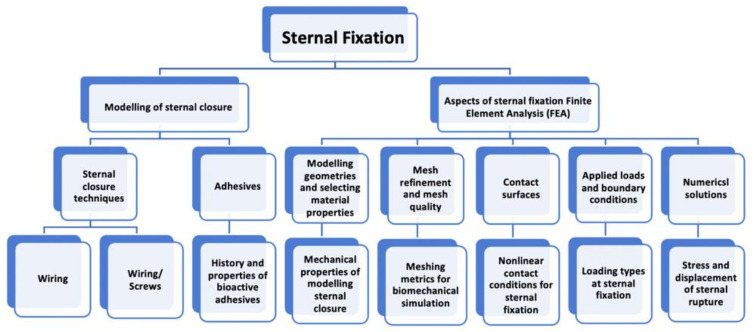
Topics that will be reviewed for modeling the sternal fixation.

Testing a bioactive material can predict the potential success of its use in the modeled median sternotomy. Further, studying the physical behavior of the 3D sternal fixation models numerically will reduce the cost of running clinical experiments. However, to date, there are no numerical modeling studies that investigate the use of different sternal wiring closure techniques in conjunction with adhesives. Thus, the aim of this paper is to review numerical and biomechanical modeling studies of sternal fixation. To achieve this, a review of common sternal closure techniques, adhesives for sternal closure, the biomechanics of modeling sternal fixation, and the finite element modeling of sternal implant systems will be presented. Figure 1 shows the topics that will be reviewed in this paper in more detail. The discussion of the literature on these topics will be useful in assessing the effectiveness of simulating new sternal fixation models.

## 2. Relevant Anatomy of Median Sternotomy

Martini et al. [18] stated that the human thorax is composed of the sternum, the ribs, and the synchondrosis cartilaginous connections between the upper ribs and the manubrium. The internal organs within the chest are protected by the sternum and ribs. The sternum, located anteriorly in the middle of the chest [19], serves as a support element of the respiratory thorax [20]. Further, the correlation between the sternum, ribs, costal cartilages, and intercostal muscles facilitates the motion of the chest wall [21]. The sternum tissue contains calcium ions and collagen connective tissues, which help to prevent bone fractures from occurring within the body. The bones are strengthened by calcium and elasticized by collagen [19]. Bones are composed of internal and external structures. The outer structure of bone is composed of cortical bone. The cortical bone mainly forms the endosteum, while the inner structure of cancellous bone forms the bone marrow [18,19]. 

The sternum consists of three sectors: the manubrium, body, and xiphoid process [18]. The manubrium is the uppermost sector of the sternum. It is widest at the top and narrows at the point where it meets the sternal body, resembling a triangular shape [22]. Dasika, Trumble, and Magovern [23] proved in their study that the manubrium is the most stable sector of the sternum. The middle, and longest, sector of the sternum is called the body. The body broadens at the bottom, where the xiphoid process connects. The lowest and smallest sector of the sternum is called the xiphoid process, which is located inferiorly [23]. In 1954, Stewart [24] specified the joint connections of the sternum. He also stated that certain bone areas adjacent to sternum joints show a definite sequence of modifications throughout ages.

Muscles connected to the xiphoid process include the diaphragm and rectus abdominis. Respiration allows the lower thoracic diaphragm to move at a greater distance than the upper thorax [19]. This causes a greater force on the xiphoid process than the rest of the sternum. The lower thorax also moves at a greater distance due to the smaller dimensions of the xiphoid process in comparison to the manubrium [19]. A test by Dasika, Trumble, and Magovern [23] indicated that the difference in sternal closure forces is most evident at the lower part of the sternum, meaning the xiphoid process is the most sensitive sector during sternal closures.

Median sternotomy followed by sternal fixation can cause complications such as sternal displacement. Moreover, infection may occur, which can cause pain and pulmonary dysfunctions [7]. Hence, studying sternal fixation numerically is needed to investigate the mechanical performance of sternal closure models and prevent sternotomy complications. The following section will review the sternal closure modeling to determine if these closure strategies can be used clinically in future investigations.

## 3. Modeling Sternal Closure

### 3.1. Sternal Closure Techniques: Wiring and Adhesives

An ideal sternal closure should provide stability and a low rate of post-operative problems [25]. Hence, the following section reviews the common sternal fixation techniques and adhesives discussed in cardio-surgical guidelines for sternal closure. 

#### 3.1.1. Wiring

The most common sternal closure technique is wiring. Robicsek et al. [25] reported that stainless steel cerclage wires are the most well-known and commonly used sternal fixation tool despite their instability under different physiological loads. Elfström et al. [26] evaluated the most common sternal closure styles numerically using finite element analysis. It was stated that the most common wiring techniques are alternating trans-sternal wiring (wire sutured loops within the sternal body) and peri-sternal wiring (wire sutured loops around the sternal body, passing between the ribs) [26]. Using a combination of alternating trans- and peri-sternal wiring has demonstrated superior strength and stability in finite element analysis due to its simplicity and affordability. However, the wiring method has disadvantages related to re-opening of the sternum due to post-operative bleeding [27]. It is difficult to replace the wire due to excessive pain or wire degradation after re-opening the sternum. Further, wires can easily injure osteoporotic bone and penetrate it by causing a tear medially [26].

Another wiring closure style is known as the ‘Figure-of-eight’. This technique can be performed successfully; however, it should be used with caution as large areas of the sternum may be exposed if any figure-of-eight wire fractures or breaks apart [28]. Furthermore, the propensity for the failure of sternal wire closure depends on the number of closed turns and its plastic deformation [29]. Capek et al. conducted computational and experimental trials varying the number of sutured wire turns to predict the sternal fixation strength [28]. They found a proportional relationship between the number of wire turns and the failure force [28]. As the number of turns in the twisted wire increases, the failure force in the wire increases, causing a higher incidence of wire fracture. The optimal wiring techniques should provide bone stability and reduce sternal displacement [28]. Moreover, Casha et al. [29] revealed the disadvantages of applying an excessive number of turns for sternal wiring closure. The maximum strength of sternal wire and the rigid physical characteristics of sternotomy closure has been studied. The wiring closure was investigated by changing the number of twists in a steel sternal model. The correlation between the number of twists versus the maximum closure strength and rigidity was tested using the Pearson correlation coefficient. Relationships between closure rigidity, test load and number of wire twists were found using regression analysis [29]. Maximum rigidity was obtained for two-twists and declined from three to ten-twists. The results parameter as closure rigidity. Casha et al. [29] concluded that the ideal number of wire twists must be kept at a minimum to increase the rigidity of the sternotomy closure and enhance bone healing. Excessive number of twists should be avoided, as this leads to the weakening of the closure and an increase in the amount of external material in the wound thereby increasing the risk of wound sepsis [29]. Further, Shafi et al. [30] examined the effect of single wire compared to figure-of-8-wire in high-risk groups (e.g., obese patients) and found that the sternal stability was higher in single wire vs. figure of 8 wire closure technique. 

Fedak et al. [31] indicated that complications of the wiring technique arise when using standard wiring for sternal closure. Although wire cerclage is the standard technique for sternotomy [31,32] and has been proven to prevent sternal displacement [31], distracting physiological forces such as coughing result in pathological sternal displacement. Post-sternal complications may occur if the sternal displacement exceeds 2.0 mm [31,32]. Subasi et al. [33] numerically analyzed the stress reduction in the cortical bone of the sternum when the steel wire cerclage changed. The numerical simulation results of this study showed that biocompatible elastomer (Pellethane) coating wire is more effective in reducing cortical stress than AISI 316 L stainless steel wire. However, the addressed sternal cut may experience complications [34]. Thus, standard wiring is not the optimal solution for sternal fixation. Table 1 shows the different wiring closure styles and the significance of these wiring styles. 

The sternum with the added wires or screws is studied numerically to investigate the redistricting forces that maintain the coaptation of the cut edges of the sternum. Jutley et al. [34] constructed a finite element model of a sternum that had a cortical shell surrounding the cancellous bone, with a screw/wire passing through the sternum. The force across the sternotomy was calculated as T=RLP , where T is the force applied on the wire or screw across the sternum, *R* is the radius of the sternum, assuming it is a cylinder, *L* is the length of the sternum, and *P* is the pressure applied. In this study, the radius of the modeled sternum cylinder was 0.15 m, the length of the chest was 0.25 m, and the pressure corresponding to cough was assumed as 40 kPa. As a result, the calculated force across the sternum was 1500 N. This force was divided into 6 forces for each of the wires or screws (250 N). For simplicity, a small block section of the sternum was simulated computationally. When comparing screws with wires alone in the closed sternotomy, the screws reduced the contact stress by one-seventh compared to the wires alone. Hence, it was concluded that the screws prevent sternal dehiscence following heart surgery [34]. In addition, De Cicco et al.’s [41] study revealed similar results. The study indicated that cannulated screws show effective stabilization of standard sternal closure in patients with a high risk of sternal dehiscence.

#### 3.1.2. Adhesives 

In this section, a comparison of different biomaterials will be presented, along with the criteria required for bioactive adhesives. Evaluation of the appropriate bioactive adhesives allows the determination of the osteoconductive and sealing properties and selecting the proper adhesive with wiring for sternal fixation modeling. Mehrvar et al. [42] indicated that wiring without adhesives was the standard treatment preference for sternal fixation. However, recently, fixation with adhesive-enhanced wiring has been studied to assess sternal fixation stability. The position stability of bioactive adhesives between the sternum halves is crucial for rigid sternal fixation [36]. Fedak et al. [31] examined the Kryptonite biocompatible adhesive enhanced sternal closure in a randomized controlled trial and observed improvement in post-operative sternal closure. Moreover, Mehrvar et al. showed that glass polyalkenoate cement (GPC) is a potential adhesive for sternal fixation. Adding this adhesive to standard wiring can enhance the fixation closure by reducing the pathological sternal displacement [43]. Moreover, bioactive adhesives work effectively in repairing bone defects in various bone applications such as facial skeleton repair, oral surgeries, and calvarial defects [16]. The novel biocompatible bone adhesives (Kryptonite, Callos, and GPCs) are reviewed below in more detail. 

The first significant bioactive adhesive is Kryptonite, a biopolymer derived from castor oil [40], which is a better retrograde filling material than amalgam because of its osteoconductive properties and sealing ability. This biocompatible cement was used to repair oral defects; however, it undergoes volumetric expansion as it sets during the curing process [44]. In addition, Lim et al. identified Kryptonite (Doctors Research Group Inc, Southbury, CT, USA) as a bioactive bone cement for long bone fracture fixation [45]. Moreover, Fedak et al. [31] found in 55 patients undergoing primary sternotomy that Kryptonite enhanced functional closure recovery. No adverse complications were observed after 12 months, and incisional pain was reduced in conventional wiring with adhesive-enhanced closure patients. This examination was also suggestive of sternal healing in high-risk patients [31]. However, other studies suggest that applying Kryptonite in conjunction with stainless steel sternal wires limits the sternal displacement [31,32]. Further, Doumit et al. [46] declared that Kryptonite might be associated with medical complications, such as post-operative numbness, due to its unpredictable expansion properties. Thus, the product is no longer available, and newer products should be developed [45,46]. 

Pradeep A et al. [47] reported recent advancements in the management of sternal wound infection following heart surgery and stated that mediastinitis was reduced by applying materials that control bleeding and promote sternal healing. One of these potential sternal adhesives is Callos^®^ (α-tricalcium phosphate, calcium carbonate, and monocalcium phosphate (MCPM)). Muehrcke et al. [48] investigated the hemostatic effect of Callos^®^ and found that the bleeding was halted immediately when Callos^®^, an FDA-approved calcium phosphate cement, was placed on the sternal margins of 246 patients. They also looked at the impact of Callos^®^ on bone repair in 18 patients with osteoporotic sternums. The use of Callos^®^ was proven to improve sternal healing and bone growth while also allowing for total absorption of the substance [48].

Some bone models react negatively to some adhesive formulations of calcium sodium phosphosilicate [17]. Kobayashi et al. investigated three formulations of calcium sodium phosphosilicate in a sheep vertebral bone model. The histological properties were evaluated after 0, 6, and 12 weeks. They found bone resorption and inflammatory reactions when bioactive formulations of calcium sodium phosphosilicate were introduced as putty in sheep bones. Although the degree of inflammation severity decreased between weeks 6 and 12, there is a crucial need for an optimal bone graft substitute [17].

Glass polyalkenoate cements (GPCs) have been introduced as alternatives to bio-adhesive such as Kryptonite and other formations of calcium sodium phosphosilicate for sternal fixation augmentation [49]. In 1971, Wilson and Kent developed GPCs as dental materials [45,50]. Moreover, Chung et al. [51] conveyed that GPCs have potentially superior fixation outcomes over the volar locking plates. In 2021, Panagiotopouloou et al. confirmed that adhesives had been proposed to treat bone fractures; however, these adhesive materials have not been used as a treatment for bone fixation in clinical practice [50]. 

Chemically, a silicate-based glass of different particle sizes and polyacrylic acid of different molecular weights are set in de-ionized water to make GPCs [52]. In 1994, Darling and Hill [53] reported that GPCs release ions such as calcium (Ca) and zinc (Zn) which promote faster bone healing. Furthermore, Inzana et al. [54] developed the formulation of calcium phosphate and calcium sodium phosphosilicate in collagen scaffolds and proved that it maximizes bone healing and assists bone regeneration. These formulations of GPCs were implanted with or without autografts when injected for femoroplasty [54]. GPCs can also sustain the growth of calcium phosphate layers at their surface [16]. Clarkin et al. [16] indicated that GPCs based on strontium exhibit potential handling properties and mechanical strength that allow the support of the trabecular bone. Marx et al. [55] suggested that the presence of strontium (Sr) released locally from GPC is enough to promote bone formation and accelerate the healing process. Wren et al. [56] indicated that the integration of Sr in GPCs is recognized to improve bone-forming properties without hindering the strength of the cement in orthopedics. This cement eases the formation of amorphous calcium at the cement surface. This formation speeds up the coverage as it increases bone density. 

The adhesive cement selected for modeling should be an adhesive that maintains certain biological and mechanical criteria to understand its performance that may resemble outcomes of real clinical trials. The bioactive cement criteria are necessary for efficient sternal fixation treatment. Table 2 identifies the biomechanical properties and the preferable endpoints for testing the sternal fixation. 

The best adhesive model should result in low stress in the designed treated area. Furthermore, when the geometry of the modeled adhesive differs from the specified sternal model, different outcomes are obtained. Sukumoda et al. [59] indicated that increasing the adhesion area reduces the risk of debonding, therefore, reducing stress on the adhesive layer. GPCs are bioactive in nature; thus, they can be modeled to understand how they work in the designed sternal fixation. Khader et al.’s [60] study analysis showed GPCs as potential bio-adhesives due to their significant mechanical properties. Moreover, bio-adhesives are recommended because of their chemical adhesion to bone [61], radiopacity [57], and ease of application [16]. Thus, the review indicates that GPCs have advantages over conventional fixation techniques, and their mechanical performance can be investigated numerically in future studies with the aim of using them clinically in sternal fixation.

### 3.2. Finite Element Models 

Finite element analysis (FEA) simulates the mechanical behavior of orthopedic designs under different loading conditions and can be a very significant tool for the assessment of biomechanics in orthopedics [62]. The evaluated results are used to optimize design and treatment effectively [62]. FEA has been used in applications such as determining feasible prostheses to support bone with large defects [63], predicting bone strength [64], testing the quality of bone after treatment [65], and studying dental implants [66,67]. FEA has also been used to evaluate design models of chest bone parts and customized implants [68]. In a study by Manić et al., the sternum bone model was created from virtual images of a healthy sternum model. The 3D geometrical model was analyzed to generate the dimensional constraints and characteristics of the sternum cross-section to support it with customized implants [68]. In addition, FEA allows for evaluating the biomechanical performance of the sternal fixation techniques. For instance, Ni et al. [69] used FEA to model clavicle–sternal fixation methods. The implants used were evaluated numerically by studying the stability, fracture micro-motion, and stress distribution of the constructed model.

**Figure 2:**
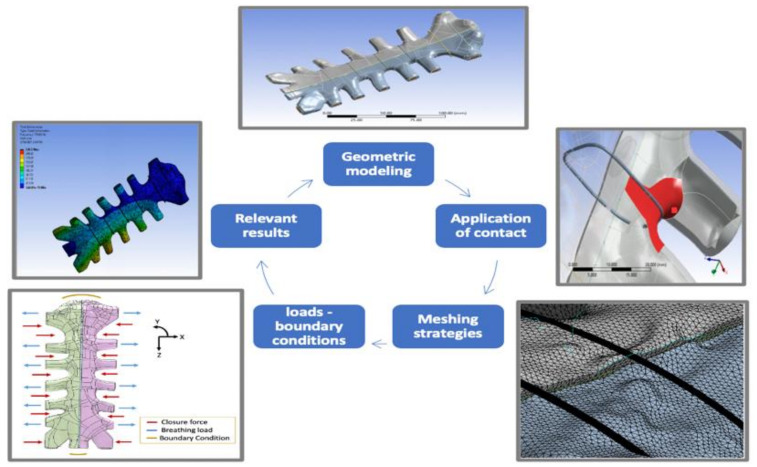
The finite element steps for simulating sternal fixation models.

In recent years, computational analysis has been increasingly applied to assist clinical technologies for cardiovascular operations [70,71]. FEA can be used to perform structural analysis of complex sternal closure techniques. However, Schimmer et al. [72] stated that biomechanical studies had not reached a consensus regarding the best sternal closure model and the efficiency of different sternal closure techniques. FEA can be developed to evaluate the complications and provide analysis to prevent mechanical failure [73]. Auricchio [74] reported that the finite element method could be used to test designed structures of specific material properties based on certain applied mechanical loads. The steps required to numerically simulate the static structural behavior of a sternal fixation for biomedical studies are summarised as identification of the geometric modeling, material properties, meshing techniques, contact conditions, loads applied, and the numerical solutions. Figure 2 shows an overview of the FEA steps in studying sternal closure.

The simulation process begins with the geometric modeling to be examined using finite element analysis. The simulation of sternal fixation includes three geometries: the sternum, wires, and adhesives. Second, the material properties are identified for each part to test the modeled material numerically and predict results to experimental conditions. Consequently, changes in the modeling processors are applied to accommodate the linear elastic model of the bone. Third, meshing techniques are selected based on the size and complexity of each part. The meshing technique determines the number and correlation of elements in the geometry. Fourth, the contact types between the geometrical parts are selected to identify the resembled clinical conditions. To preprocess this step, the formulation type and behavior of each contact type must be identified in order to adopt changes between the parts in the system. Fifth, the various loads are applied to create real-life clinical scenarios in a virtual mechanical environment. Last, the numerical solutions are analyzed to solve the modeled system and carefully examine its behavior under different conditions. The next sections will review in detail the steps required to numerically simulate the sternal fixation model.

#### 3.2.1. Geometric Modeling and Material Properties

To model the sternum numerically, the 3D geometry of the sternum should be mapped in the simulating program to mechanically study its behavior under certain loading conditions. Selthofer et al. [75] studied the morphometry of the sternum. The breadth, length, area, and thickness of the sternum segments were analyzed for 35 females and 55 males with an average age of 65 years to evaluate the standard shape of the sternum. After measuring the sternum parameters for 90 human sterna, they found that the sternum has a non-standard shape. Accordingly, researchers have investigated simpler methods to model the sternum by either artificial materials such as polyurethane foam [76] or by using CT scans [76,77].

After developing the geometry, the material properties are determined for the simulated 3D model of the tissue to generate accurate solutions [62]. The most important properties for this simulation are density, Young’s modulus, Poisson’s ratio, thermal coefficient factor, and tensile strength of the material. Modulus of elasticity (Young’s Modulus) is one of the material properties that is needed for the linear elastic model of the bone. It determines the bone’s ability to resist changes in geometrical length as a result of tension or compression. Abendschein & Hyatt [78] explained that when the bone is attached to other orthopedic implant materials, it produces intermolecular resistance to the deformation generated by the external force. The bone, however, is heterogeneous and viscoelastic as it is a composite of collagen and apatite. Previous studies revealed that the Young’s Modulus value of cortical bone is different from the cancellous. Table 3 summarizes the values commonly used to numerically model the behavior of sternal bone.

#### 3.2.2. Mesh Refinement and Mesh Sensitivity

In FEA, meshing refers to creating a grid of connective elements to discretize a complex geometry. Mesh refinement is an important step in an FEA simulation as it simplifies irregular shapes into more recognizable elements for FEA solver software [82]. Banichuk et al. [76] specified that mesh refinement gives better quality of FEA results. Zhang et al. [79] declared that general mesh refinement might not be sufficient for a simulation to converge; hence local mesh refinements are required. Local mesh refinement is considered one of the important implementation steps of adaptive FEA. The adaptive FEA is composed of two refinements types, regular refinement and local refinement [79]. Orhan and Ozyazicioglu [83] specified in their FEA of sternal closure that refining the mesh would improve the accuracy of the computation.

More and Bindu [84] studied the effect of mesh sizing on finite element analysis and indicated that mesh sizing errors have a large impact on the numerical results. The results become mesh independent when the values do not change despite the mesh refinement changes. In the end, mesh refinement is a trade-off between the accuracy and speed of the numerical simulation [84].

In an assessment of a thorax model, Cronin et al. verified that, unlike the coarser mesh, the refined mesh and the numerical contact of the thorax muscle tissue leads to a strong contact of the simulated surfaces with no interpenetration of the surfaces meshes [85]. Kashan et al. [86] studied the mechanical behavior of simulated scaffold applications and explained that mesh refinement is executed to increase the quality and accuracy of the numerical results.

#### 3.2.3. Mesh Quality (Mesh Metrics)

Checking the mesh quality is needed to eliminate an improper selection of the mesh that results in lower accuracy and longer simulation run times. To perform the right type of mesh, different metric parameters are checked. Burkhart et al. stated that the literature has not been updated with mesh metric studies in finite element modeling of bone tissue [87]. It was found that 95% of the reviewed models did not assess the mesh quality of their finite element analyses. It was also mentioned that aspect ratio, skewness, and the Jacobian rate are the mesh metrics that should be used to assess mesh quality. The following section will explain the essential mesh metrics to check the mesh quality. The standard quality parameters in modeling software are warp angle, aspect ratio, skewness, Jacobian ratio, and distortion. Table 4 identifies the mesh metrics constraints and their modeling mesh solutions to overcome any related meshing error.

Warp angle is measured only on elements that have more than three nodes. It is calculated from the element arcsin of the edge size to the height of an element to check how far it is from a planar [88]. The second quality parameter is the aspect ratio, which compares various lengths in an element. It is equal to maximum length divided by minimum length. Fellipa [89] recommended that the aspect ratio should be larger than 1 and less than 3 [89]. The third quality parameter is the skewness parameter (angle idealization), which shows how the meshed element varies from the ideal triangle element or ideal square quad element [90]. It is strongly recommended that skewness be less than 45 degrees. In the nonlinear FEA of a sternal closure, Orhan and Ozyazicioglu [83] used the skewness parameter to check the mesh quality. The fourth quality parameter is the Jacobian, which helps to identify distortion in the element. The Jacobian is a representation of the size, shape, and skew matrices of an element. Knupp [90] specified that the determinant of the Jacobian matrix plays an important role in identifying the mesh quality. It regulates the transformation of defected meshing elements to much simpler shapes. The Jacobian should be between −1 and 1. The Jacobian matrix meshing errors are critical and difficult to fix. Thus, remeshing the defective modeling parts is required. Lebschy illustrates that remeshing each segmented part of the human thorax is required in the processing of its biomedical modeling analysis [91]. The fifth quality parameter is mesh distortion. Large structural deformation causes mesh distortion, which leads to numerical issues. Distortion errors are common in meshing analysis. To reduce meshing distortions, nonlinear mechanical shape checking is used to increase the global element quality of the mesh. Increasing mesh density is recommended to overcome the excessive mesh distortion that occurs in a low-density mesh [92]. Errors in setting proper meshing may cause convergence errors in the finite element analysis. Hence, high-order meshing is necessary to solve these errors. Studying the mesh metrics is necessary to assess the mesh quality of bone models in finite element analysis.

#### 3.2.4. High Order Meshing

Henke and Shanbhag [93] reported that generating irregular meshes are desirable for efficient and versatile representation of complex geometries; however, best practices for irregular meshes are not established [93]. High-order meshing is an optimization-based mesh that smooths curved edges of elements. Persson et al. [94] claimed that one of the poorest solved meshing issues is generating high-order meshing for elements that conform to the boundary geometry. Small isotropic elements in contact with boundary geometries are easy to deform, but larger elements fail to deform. Hence, the meshing strategy is not processed successfully. Therefore, a sufficient finer mesh for large elements in contact with the boundary guarantees resolving the deformation of non-intersecting elements in highly distorted meshes [94].

#### 3.2.5. Mesh Methods

Xiao et al. [95] reported that the accuracy and efficiency of meshing approaches depend on the discrete representation of the geometric model. In addition, Henke and Shanbhag [93] indicate that meshing methods are approached to record the deformation in continuum and discontinuous segments that develop during the simulation.

Various meshing types are required to enhance the computational results and accommodate the complex geometry. In addition, for more accurate and convergent results, various meshing sizes are required. Studying the mechanical behavior of biomedical implants such as hip implants using finite element analysis needed investigation of various meshing strategies to accomplish accurate convergence results [96]. Therefore, adaptive meshing is added to adjust the complexity of the geometry. For a better meshing quality in a biomechanical study, selecting multiple meshing types is important for reliable results.

There are several advanced meshing techniques used for simulating complex systems [97]. The tetrahedral meshing method is better than the hexahedral meshing method because it is easier to mesh and predict element transitioning in complex geometries [97]. The algorithm for the tetrahedral meshing method can be either patch-independent or patch-conforming. The patch-independent algorithm leads to an excellent skewness and aspect ratio of the elements. Hence, the patch-independent algorithm is preferred for bioengineering simulations as the meshing is done for a smaller number of cells in less computation time [82].

In the tetrahedral meshing method, the patch-independent algorithm is superior, as it is good for gross defeaturing CAD geometries, while the patch conforming algorithm is recommended for clean CAD geometries [97]. The meshing criteria of patch-independent for tetrahedral elements work better than patch conforming for testing the mechanical behavior of the sternal fixation. Orhan and Ozyazicioglu [83] selected a 10-node tetrahedral element for meshing the sternum with the closure material as it was proven to provide the suitability of these elements in complex geometries. Hence, the meshing criteria of the patch-independent technique make it a better selection for the tetrahedral meshing method. This gives more reliable results for testing the mechanical behavior of the sternal fixation. Kashan et al. [86] explained that mesh refinement is executed to increase the quality and accuracy of the results.

#### 3.2.6. Contact Surfaces

Contact surfaces between parts of the simulated model should be selected to present the most reliable numerical study. Reliable contact settings occur when compressive or tensile normal forces are transmitted without causing penetration in the system [94]. Bonded contact does not allow any sliding or movement at the interfaces. However, non-linear frictional contact allows sliding and movement between surfaces. This movement is restricted by the coefficient of frictional sliding. Lim et al. conducted a numerical study where bonded contacts were set between the sternum and ribs to prevent their separation [81]. Generally, the aim of selecting the best contact type is to prevent separation or excessive penetration in the system. Table 5 summarizes the difference between the contact types and their specification for use in ANSYS software, which is used for finite element analysis.

The contact formulation of pure penalty presents the contact as force, in which the normal stiffness is multiplied by the penetration length. However, the augmented Lagrange formula has an extra term of penetration length in another degree of freedom which makes it less sensitive to the stiffness factor [99]. In a contact pair, the pure penalty contact type can work better for a simple simulated case as it minimizes numerical contact issues such as the occurrence of geometries penetrating each other at the contact surfaces. The detection method should be at nodes of the contact surface with a direction perpendicular to the target surface. When the contact elements touch target elements after applying the reaction force, the pure penalty type is selected to build up the proper sliding between the surfaces. Thus, a penalty-based contact type is selected for simulating the sternum and closure tool.

Nonlinear contact conditions are commonly used for studying complex models. In 2019, Orhan and Ozyazicioglu applied nonlinear contact conditions to evaluate sternum closure techniques [83]. Frictional contact was modeled between the sternum halves, and between the sternum and the closure material; the contact formulation selected was Augmented Lagrange. Using nonlinear contact requires the stiffness factor for the contact to be reduced to a value between 0.1 and 0.05, depending on the bioactive material used and how the model behaves. These contact settings are expected to work better with a finer mesh.

#### 3.2.7. Applied Loads and Boundary Conditions

In a numerical simulation of sternal fixation, loads and boundary conditions are applied to the sternal model to evaluate the movement of the sternum in the thoracic system under physical conditions. There are several forces that act on the sternum, such as the breathing force, the force of laying down on one side, and the force of arm movement. This section reviews different kinds of loads and how they are applied in the sternal system for both physical experiments and numerical simulations. Cohen and Griffin [100] indicated that anterior–posterior shear was applied to the sternum to present the breathing and coughing loads, whereas cranial– caudal shear was applied to resemble the sternum movement when hands are moving. Moreover, Losanoff et al. [28] performed uniaxial testing where the sternum was laying vertically, and perpendicular force loads of (0–800 N) were applied for 40 minutes to measure lateral stresses on the adult human cadaveric specimen, and failure of sternal closure during this period. The predominant lateral stress occurred during breathing, but the sternum was not stressed by minimal force loads in the anterior–posterior and rostral–caudal directions.

Another loading technique was used by Gunja et al. [101]. In this study, sternal fixation was evaluated by applying lateral load to measure the sternal distraction between the two sternal halves. Average distractions at different locations of the sternum were calculated. A tensile test was conducted to determine the stability of three plate models at the sternum. The rigid plated sternum was laterally pulled to a maximum load of 400 N to measure the stability of the model [101].

In another study, Saito et al. conducted biomechanical experiments where loads were applied as shear stress in an anterior–posterior direction and in a cranial–caudal direction to predict if the bioabsorbable Poly-L-Lactide (PLLA) sternal pin prevented the displacement of the sternum in both directions. The fixation stability was evaluated using the stiffness defined as the slope of load applied over the outcome measured displacement of sternal halves [102]. The results of applying the shear stress load in an anterior–posterior direction and cranial–caudal direction showed that the addition of a PLLA pin to steel wiring reduces the stiffness and provides adequate sternal fixation [102].

**Figure 3:**
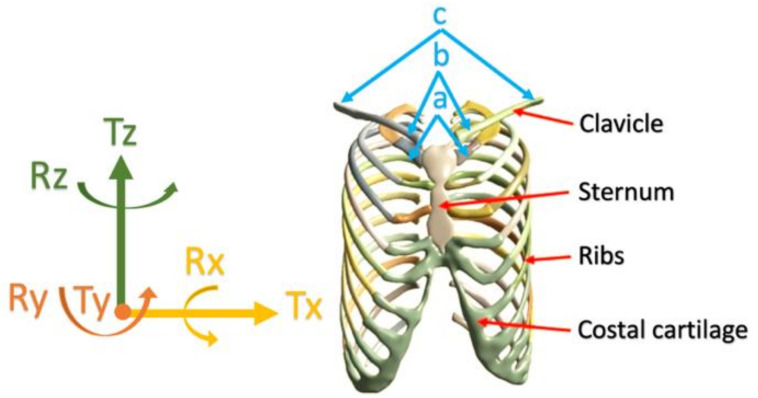
Redesigned representation of the load’s directions and locations of boundary conditions around the sternoclavicular joint [80].

Lim et al. conducted modeling studies to examine the effect of Pectus excavatum (sunken chest) on the sternoclavicular joint. Figure 3 was remodeled as an illustration of the sternum in the chest and the directions of displacement and rotations of the sternoclavicular joint. The FEA model was constructed to test the following six cases [80]. Loads and boundary conditions are explained according to Figure 3, where point ‘a’ refers to the sternoclavicular end between the sternum and ribs. Point ‘b’ refers to the acromial ends, and point ‘c’ refers to the clavicle ends.

(1) ‘c’ ends are fixed, ‘a’ ends are unconstrained, allowing movements in all directions;
(2) ‘c’ ends are fixed, ‘b’ ends are constrained with the sternum by bonded contact;
(3) ‘Tx = 0, Ry = Rz = 0′. Joints ‘a’ translation is constrained in *x*-axis and rotations are constrained in *y* and *z* axis;
(4) ‘Ty = 0, Rx = Rz = 0′. Joints ‘a’ translation is constrained in *y*-axis and rotations are constrained in *x* and *z* axis;
(5) ‘Tz = 0, Rx = Ry = 0′. Joints ‘a’ translation is constrained in *z*-axis and rotations are constrained in *x* and *y* axis;
(6) ‘Tx = Ty = Tz = 0, Rx = Ry = Rz= 0′ the cartilage joints were set as fixed condition.

On the other hand, Orhan et al. pre-loaded the sternum with different types of loads (lateral distraction, longitudinal shear, and torsion). They conducted a study of four sternum closure techniques to determine the deformation modes. As Table 6 shows, the lateral distraction load was at the highest allowable level when it gave the minimum rupture displacement. However, the sternal fixation was weaker at resisting the longitudinal shear and torsion. As a result, the critical displacement was 19.6 mm [103].

Orhan and Ozyazicioglu, in another study, evaluated sternum closure methods under lateral distraction loading by a nonlinear FEA [83]. The allowable loads applied in this study are listed in Table 7.

The sternum was pre-loaded to the max with additional wires to separate up to 2.0 mm apart. Additional wires were applied to modify the closure technique. The load required to reach 2.0 mm displacement along the incision area was 2380 N [82].

Further, Fawzy et al. [104] examined the sternal stability at the primary and secondary sternal closures by the plating and wiring methods. The loads were applied as intrathoracic pressure to evaluate the sternal stability. Sternal separation was measured when intrathoracic pressure was increased to reach the longitudinal separation required (2.0 mm), which is clinically the separation before any significant damage to the sternal bone. Table 8 summarizes the loads reviewed in this section to facilitate the understanding of the various loading types for sternal fixation modeling analysis.

The current mechanical studies’ literature evaluation reveals that several loading strategies can be used to model the forces operating on the sternal fixation models. The two most commonly used loading types are the longitudinal shear, which represents the breathing force, and the lateral load, which represents laying down. These forces are applied to determine the stability of the sternum prior to sternal separation. Mechanical investigations of the sternal closure show that the maximum loading values vary from one experiment to another based on the material constitutive model. The mechanical studies found that the measurement of the least separation between the sternal halves before any sternal rupture is (2.0 mm). Corresponding loading values that can be implemented for modeling sternal fixation would be determined based on the selected sternal closure technique.

### 3.3. Numerical Solutions and Evaluation of Modeled Sternal Fixation Systems

There are a limited number of studies that use numerical simulation to compare sternal fixation using various metal closure tools. The complex sternal fixation models discussed in this section are summarized in (Table 7). The reviewed literature focused on evaluating the dynamics of different wiring techniques [26] or modeling the sternum following median sternotomy without wire closures [28]. However, no finite element study has been performed to analyze the sternum with wires and adhesives in one 3D model. In this section, a summary of previous studies is presented to demonstrate the difference in 3D modeling techniques of different sternal closure studies and assess them by FEA.

Jutley et al. [34] simulated a segmented block of the sternum with wires and screws that go through it to measure the contact stress exerted by the cough force and the wire force as specified in Section 3.1.1. The direction of the force in this block model was subjected along the wire/screw and bone interface to close the sternum. The meshing distribution was refined at elements close to the wire. Jutley et al. found that the contact stresses of the closed median sternotomy are high in thin cortical shells. In addition, results showed that using six screws decreased the contact stress within a few millimeters around the area of the screwed bone [34].

Burkhart et al. [87] reviewed the finite element models of bone tissue to study the mesh quality and associated validation methods. The paper claimed that 42% of the finite element models were not validated adequately [87]. Most documented studies focused on evaluating the dynamics of the sternum with wires only. The following studies are reviewed to evaluate the technical methods used for simulating sternal closure models. Trumble et al. [105] studied two frequently used wire closure techniques. These techniques were tested using artificial sterna models created from solid polyurethane foam and whole cadavers. The sterna models were formed using polyurethane foam (320.39 $\mathrm{kg}/m^{3}$) to replicate the mechanical properties found in the human cadaveric sternum. Closures were stressed by the lateral traction force of the lungs, which presents the force of the sternal cohesion. Separation of the incision site was measured at the manubrium, mid sternum, and xiphoid. Bench tests of artificial sterna demonstrated minimal sternal separation compared to tests of cadavers. The demonstrated data of human tissue is more variable than the data of sternal models. This suggests enhancing the tests to detect minor differences in sternal fixation stability [105].

More studies have been reviewed to cover the limitation of studying loading conditions of sternal model fixation models numerically with different loading conditions. Bruhin et al. [106] studied the structural response of a human median sternotomy. Image processing methods were used to segment and analyze CT scans of the thoracic bones, and then a linear elastic material was used as a constitutive material for the bone. The thoracic scans were then transmitted to a 3D finite element model. Nonlinear contact settings were applied between the sternum fixation wires and the two sternal parts. The prescribed rotation angles at the spinal ends of the ribs were changed to examine three loading conditions: normal breathing, lateral bending, and dorsal bending of the spine. The stress response and displacement were predicted for two closure techniques (single loop and figure of eight) [106]. The results for normal breathing load cases revealed that the sternum was clamping adequately in the single loop technique. In comparison, the figure of eight loop closure technique can considerably reduce the relative longitudinal displacement between the two sternal parts when load conditions are applied [106].

Moreover, Orhan and Ozyazicioglu [83], in 2019, studied a finite element analysis of the sternum closure model based on CT images of a cadaveric specimen. However, their 3D modeled sternum was assumed to be an isotropic bilinear-elasto-plastic material. Three types of wires were used; steel bands, steel wires, and ZipFix bands. Lateral distraction loading was applied, and displacement at the incision was obtained. The analysis of the results showed that steel or ZipFix bands are superior in the sufficiency of closure techniques to conventional wiring [83]. Table 9 summarizes the complex sternal fixation models reviewed in this paper.

The numerical validation of the presented studies proves that the closure techniques were effective and did not lead to failure in wires used after insertion. However, under certain loads post sternotomy, complications occurred, such as the dislocation of wires and separation of sternum halves [48,105]. Moreover, the FEA of different sternal closure models was performed at different loading cases [101,102,103,104,105]; however, there is a limitation in studying the numerical simulation of sternal fixation using adhesives along with metal closure tools. Experimentally, after skeletal repair, adhesives proved to limit some of the bone defects, such as the inflammation severity and aspects of resorption [11]. However, the up-to-date literature is missing studies about the finite element analysis of bioactive adhesives that have been proposed in vivo for sternal fixation clinical studies. This indicates that more FEA studies of sternal fixation with adhesives are needed to analyze the efficiency of this sternal closure technique numerically.

### 3.4. Limitations of FEA in Studying Sternal Closure

There are a few major limitations of FEA in studying sternal closure techniques. First, this process is limited by sternum geometric variability but not by the effect of comorbidities (osteoporosis, diabetes, calcium metabolic disorders, etc.) or tissue materials (collagen or plasma). Second, the FEA physical and structural analysis varies based on the designed sternal model. Further, due to the variance in irregular geometries of closure techniques and variance of meshing refinement techniques, it does not produce default results. Third, it analyses the adhesive based on its mechanical properties (e.g., density and youngs modulus), not the biocompatible properties (e.g., chemical composition and ability to be completely absorbed by the bone). In general, FEA fits into the larger study of sternal closure methods. It can be a method that is used after animal studies and biomechanical testing to simulate the clinical setting before human studies.

## 4. Conclusions

Sternotomy is an invasive procedure that occurs through conventional surgery [107]. To limit the complications of this invasive procedure, advanced sternal closure techniques are required. In this literature review, several sternal closure techniques were reviewed, including wiring, wiring screws, plates, and adhesives. Alternating Peri-sternal and Trans-sternal wiring techniques proved to have greater strength and stability than other wiring closure tested methods [29]. Thus, this mechanical method can be the optimal selection for FEA modeling. In addition, GPCs have been reviewed and proposed as adhesives for sternal fixation as they possess bioactive properties and are associated with good clinical outcomes. Furthermore, there are limitations to the commonly used sternal fixation techniques. The in vivo experiments are time-consuming and costly for sternal models, but they are very effective in predicting results in the clinical setting. Hence, researchers decided to investigate the efficiency of sternal fixation techniques using computational methods such as FEA of simulated sternal fixation models. In order to have the most reliable numerical study of the sternal fixation model, mechanical properties, meshing techniques, contact surfaces, and loads should be identified for all modeled parts to reduce the simulation convergence challenges. Many studies of bone tissue modeling are not adequately validated because the mesh metrics in these studies were not reviewed sufficiently [70]. Therefore, checking the mesh quality is essential to eliminate FEA solver issues. Furthermore, refining the mesh is necessary to generate a better FEA mesh sensitivity analysis. The reviewed studies showed different loading techniques that were, in general, describing normal breathing and movement of the body in lateral and dorsal directions to determine the relative displacement of the sternal parts.

Based on the literature review conducted, using adhesives in conjunction with wiring gives the optimal results of sternal fixation [108] as the potential bio-adhesives contribute to limiting the complications of post-sternotomy [16, 60]. However, this was not reviewed numerically by means of FEA. Therefore, to cover this gap in the literature, a precise finite element analysis of 3D modeled sterna with wires and adhesives is needed. Future numerical modeling studies will have to address the finite element analysis of sternal fixation with adhesives to investigate numerically the mechanical performance of adhesives in sternal fixation. This will provide computational results that will facilitate the understanding of the sternal fixation models under different clinical conditions.

## Figures and Tables

**Table 1:** Illustration of different wiring closure styles.

Wire Closure Style	Modeled Illustration	The Significance of Wiring Style
Alternating Peristernal and Transsternal	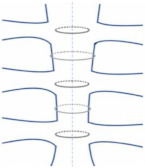	It has been the best closure technique due to its superiority in mechanical stability and strength [27].
Single Transsternal	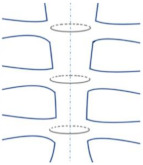	The twisted free ends of the wire may penetrate the sternum (due to osteoporosis or other factors) when wire loops are installed trans-sternaly in weak bones [28,35].
Single Peristernal	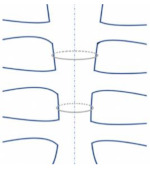	Reduces risk of deep sternal wound infection by reinforcing the corpus sterni of the sternum [36] Safe for solid internal fixation [37]. Sternal stability was higher in single wire vs. figure of 8 wire in high-risk obese patients [38]
Figure-of-eight	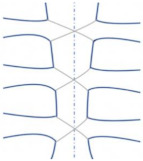	Figure of eight wires are not superior to simple wires [38,39].
Modified figure-of-eight	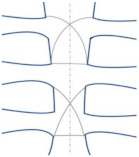	Effective and safe method for limiting sternal dehiscence by limiting the penetration in the intercostal spaces [40]
Longitudinal parasternal	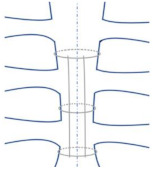	Used for high-risk patients. Prevent sternal dehiscence and sternal wound complications [11].

**Table 2:** Requirements for cements used in orthopedic medical applications.

Criteria	Required Endpoints
Radiopacity [57]	High level of radiopacity to observe sternal displacements [36]
Nontoxic nature [61]	Safe to use inside a human body
Adhesive mechanical properties [16]	Sufficient strength to withstand the maximum forces imposed during leaning on one chest side or coughing. [16,57]
Aging mechanism of the cement [57]	Sufficient working time (6–10 min) and rapid setting time (maximum 15 min)
Biocompatibility [42]	It should be a bioactive material to avoid inflammation.
Viscosity [16]	Medium viscosity is required for injection and interpenetration of trabecular spaces. [16]
modulus of elasticity	Excessive damage occurs if the elastic limit is exceeded. [58]

**Table 3:** Sternum modeling parameters for identifying biomechanical properties.

Sternum Part	Modulus of Elasticity	Density	Poisson’s Ratio	Ultimate Tensile Strength UTS
Sternum and ribs [77]	0.0121 (MPa)	----	0.20	18,000
Sternum [79]	11.50 (GPa)	$2000\;\left(\mathrm{kg}/m^{3}\right)$	0.30	----
Cortical bone Cancellous bone [34]	15.25 (GPa) 1.12 (GPa)	----	0.27 0.27	----
Cortical bone Cancellous bone [80]	11.50 (GPa) 0.04 (GPa)	----	----	----
Cortical bone Cancellous bone [81]	10.18 (GPa) 0.04 (GPa)	2000 1000	0.3 0.45	2.3 (GPa) 0.001 (GPa)

**Table 4:** Mesh metrics specs and FEA meshing solutions to their numeric errors.

Mesh Metric	Constraint	Modeling Mesh Solution
Warp angle	Elements have more than 3 nodes	Add subdivisions to elements
Aspect ratio	Element has a symmetric shape 1 < (Max length/Min length) < 3	Local mesh refinement
Skewness	Compression to ideal element	Improve surface meshing
Jacobian ratio	−1 < Jacobian determinant < 1	Remeshing defected parts
Distortion	Occurs in hyperelastic material	Increase quality of the mesh

**Table 5:** Summary of contact formulations available in ANSYS and the criteria of each contact type [98].

Pure Penalty	Augmented Lagrange	Normal Lagrange
Good convergence behavior	If penetration is too large, additional equilibrium needed	Chattering is present
Sensitive to the selection of normal contact stiffness.	Less sensitive to the selection of normal contact stiffness.	Not sensitive to the selection of normal contact stiffness.
Contact penetration is present and uncontrolled	Contact penetration is present and controlled	Penetration is almost near zero

**Table 6:** Allowable load and rupture load values of different loading types and their resultant rupture displacement values [103].

Loading Type	Allowable Load (N)	Rupture Load (N)	Rupture Displacement (mm)
Lateral distraction	1032.6 ± 120.4	1702.9 ± 327.3	6.28 ± 0.03
Longitudinal shear	579.79 ± 30.7	1458.16 ± 120.1	15.79 ± 0.05
Torsion	92.4 ± 6.44	955.1 ± 76.1	19.6 ± 0.00

**Table 7:** The load values required for different sternal closure techniques to reach a rupture displacement of 2.0 mm [83].

Load Values (N)	Closure Technique
1457	3 steel bands + 4 steel wires
1317	3 ZipFix bands + 4 steel wires
1051	conventional steel wire (7 steel wires)
2380	Additional wires at the manubrium and xiphoid

**Table 8:** Overview of the sternal loads reviewed for modeling the sternal fixation.

Reference	Load	Direction	Evaluation
Cohen and Griffin [100]	Breathing load Movement load	Anterior–posterior shear Aranial– caudal shear	Strength, stiffness, and post-yield analysis
Losanoff et al. [28]	Breathing load	Uniaxial test (0–800 N) Anterior–posterior and rostral–caudal	Lateral stress of the sternum
Gunja et al. [101]	Tensile test of sternum fixed with plates	Laterally pulled to a maximum load of 400 N	Sternal distraction between the two sternal halves
Saito et al. [102]	Shear stress	Anterior–posterior direction, and in a cranial–caudal direction	Displacement of sternal halves and fixation stability
Lim et al. [80]	External force of the raised pectus bar in the chest wall	Displacement and rotations around the sternoclavicular joint	Equivalent stress distribution on chest wall and anterior/posterior length of the chest wall
Orhan et al. [103]	Pre-loading types	Lateral distraction (1032.6 N), longitudinal shear (579.79), and torsion (92)	Rupture displacement
Orhan and Ozyazicioglu [83]	Lateral distraction loading	2380 N	2.0 mm displacement along the incision area
Fawzy et al. [104]	Intrathoracic pressure	Load increased gradually to reach longitudinal separation (2.0 mm)	Sternal stability

**Table 9:** Details of reviewed complex sternal fixation models.

Type of 3D Modeling Study	The Designed Sternal Model	Wiring Closure Technique
Evaluate stress around a ster-num screw [34]	Block of sternum model	Stainless steel wire/screw
Evaluate closure of sternum at different processes [105]	Sternal model from Polyurethane foam	single-loop vs. figure-of-eight
Evaluate structural response of the median sternotomy [106]	Cadaveric sternal CT scans modeled as linear elastic material for the bone	single-loop vs. figure-of-eight
Evaluate three sternal closure techniques [83]	Sternal CT scans modeled as isotropic bilinear-elasto-plastic material	Steel bands, steel wires, and ZipFix bands.
Evaluate strength of sternal wire and rigidity of the sternotomy closure [95]	Steel sternal model	Changing the number of sternal wire twists (0–10).

## Data Availability

Not applicable.
